# Preservation Macroscopic Entanglement of Optomechanical Systems in non-Markovian Environment

**DOI:** 10.1038/srep23678

**Published:** 2016-04-01

**Authors:** Jiong Cheng, Wen-Zhao Zhang, Ling Zhou, Weiping Zhang

**Affiliations:** 1 School of Physics and Optoelectronic Technology, Dalian University of Technology, Dalian, 116024, People’s Republic of China; 2 Department of Physics and Astronomy, Shanghai Jiao Tong University, Shanghai 200240, People’s Republic of China; 3 Quantum Institute for Light and Atoms, Department of Physics, East China Normal University, Shanghai 200241, People’s Republic of China; 4 Collaborative Innovation Center of Extreme Optics, Shanxi University, Taiyuan, Shanxi 030006, People’s Republic of China

## Abstract

We investigate dynamics of an optomechanical system under the non-Markovian environment. In the weak optomechanical single-photon coupling regime, we provide an analytical approach fully taking into account the non-Markovian memory effects. When the cavity-bath coupling strength crosses a certain threshold, an oscillating memory state for the classical cavity field is formed. Due to the existence of the non-decay optical bound state, a nonequilibrium optomechanical thermal entanglement is preserved even without external driving laser. Our results provide a potential usage to generate and protect entanglement via non-Markovian environment.

The investigation of decoherence and dissipation process induced by environment is a fundamental issue in quantum physics^1^,^2^,^3^,^4^. Understanding the dynamics of such nonequilibrium open quantum system is a challenge topic which provides us the insight into the issue of quantum-classical transitions. Protecting the quantum property from decoherence is a key problem in quantum information science, therefore a lot of effort has been devoted to develope the methods for isolating systems from their destructive environment. Recently, people recognize that properly engineering quantum noise can counteract decoherence and can even be used in robust quantum state generation^5^,^6^. Meanwhile the features of the non-Markovian quantum process have sparked a great interest in both theoretical and experimental studies^7^,^8^,^9^,^10^,^11^,^12^. Numerous quantitative measures have been proposed to quantify non-Markovianity^13^,^14^,^15^,^16^,^17^.

As a promising candidate for the exploration of quantum mechanical features at mesoscopic and even macroscopic scales and for quantum information procession, cavity optomechanical systems come as a well-developed tool and have received a lot of attentions^18^,^19^,^20^,^21^,^22^. In the theoretical research of the cavity optomechanical system, the environment is often treated as a collective non-interacting harmonic oscillators, and the quantum Langevin equations^23^ are developed to describe the radiation-pressure dynamic backaction phenomena. Significant progresses have been made in this framework^18^,^24^,^25^,^26^. Almost all of these studies are focussing on the scenario of memoryless environment. However in many situations for optical microcavity system, the backaction of the environment and the memory effect of the bath play a significant role in the decoherence dynamics^27^,^28^. Quite recently, a nonorthodox decoherence phenomenon of the mechanical resonator is also observed in experiment^29^, which clearly reveals the non-Markovian nature of the dynamics. Therefore, it is necessary to investigate the non-Markovian dynamics for the nonlinear cavity optomechanical system so that we can use the memory effects to produce and protect coherence within it.

In the following, we investigate the cavity optomechanical dynamics under non-Markovian environment and put forward a method to solve the exact Heisenberg-Langevin equations where the non-local time-correlation of the environment is included. We find that when the cavity-bath coupling strength crosses a certain threshold, the optical bound state is formed, giving rise to the nonequilibrium dynamics of the entanglement. This remarkable result indicates the possibility of long-time protection of macroscopic entanglement via structured reservoirs.

## Results

### Model

We consider a generic cavity optomechanical system consisting of a Fabry-Pérot cavity with a movable mirror at one side. The cavity has equilibrium length *L*, while the movable mirror has effective mass *m*. The cavity environment could be a coupled-resonator optical waveguide which possesses strong non-Markovian effects^30^, and the micro-mechanical resonator and its environment could be the device of a high-reflectivity Bragg mirror fixed in the centre of a doubly clamped Si_3_N_4_ beam in vacuum^29^. The corresponding Hamiltonian of the system can be written as^23^,^24^


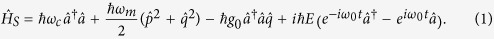


Here *ω*_*c*_ is the frequency of the cavity mode with bosonic operators 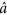 and 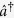 satisfying 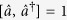, while the quadratures 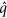 and 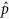 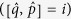 are associated to the mechanical mode with frequency *ω*_*m*_. The third term describes the optomechanical interaction at the single-photon level with coupling coefficient 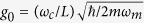. The cavity is driven by an external laser with the center frequency *ω*_0_. The environment of such system can be described by a collection of independent harmonic oscillators^31^. The reservoir as well as the system-reservoir interaction is then given by


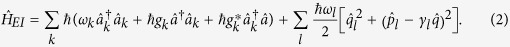


The first term is the free energy of the cavity reservoir with the continuous frequency *ω*_*k*_ as well as the hopping interaction between the cavity and the environment with the coupling strength *g*_*k*_. The second summation describes a mirror undergoing Brownian motion with the coupling through the reservoir momentum^23^,^31^. Here *ω*_*l*_ is the reservoir energy of the mechanical mode, and *γ*_*l*_ stands for the mirror-reservoir coupling.

### Dynamics of the system

To achieve a comprehensive understanding of the decoherence dynamics, one has to rely on precise model calculations. To this end, by making use of the reference frame rotating at the laser frequency, we can obtain the Heisenberg-Langevin equations


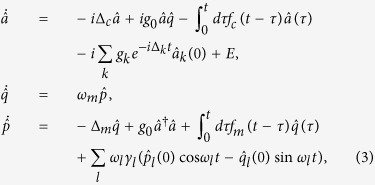


where Δ_*c*_ = *ω*_*c*_ − *ω*_0_ is the cavity detuning, Δ_*k*_ = *ω*_*k*_ − *ω*_0_ is the detuning of the *k*-th mode of the environment, and 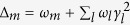 is the reservoir-induced potential energy shift. The non-Markovian effect is fully manifested in Eqs. (3), where the non-local time correlation functions of the environments 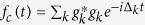 and 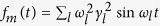 are included. By introducing the spectral density *J*(*ω*) of the reservoirs, one can rewrite the time correlation functions as 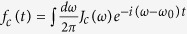 and 
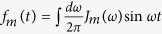
. The terms containing reservoir operators 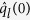, 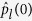 and 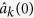 are usually regarded as the noise-input of the system, which depend on the initial states of the reservoirs.

The integro-differential Heisenberg-Langevin equations Eqs. (3) are intrinsically nonlinear. Up to now, most experimental realizations of cavity optomechanics are still in the single-photon weak coupling limit^20^,^32^,^33^,^34^, i.e., 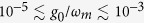. When the intracavity photon number 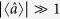, we can apply the so-called linearization method^24^,^35^, which means the relevant quantum operators can be expanded about their respective mean values: 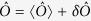, where 
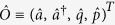
. The superscript *T* represents the transpose operation. Then Eqs.(3) can be decomposed into two parts. The first is the classical part that describing the classical phase space orbits of the first moments of operators


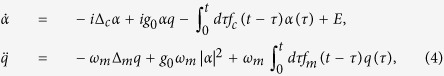


where, for simplicity, we have assumed 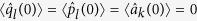. In the single-photon weakly coupling regime, the coupling strength *g*_0_ is the smallest parameter in Eqs.(4). We therefore perform the regular perturbation expansion in ascending powers of the rescaled dimensionless variable *g*_0_/*ω*_*m*_ (for computational convenience one may set *ω*_*m*_ = 1, and the other rescaled parameters are in units of *ω*_*m*_). By substituting the expressions with rescaled *g*_0_ (i.e., 
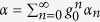
 and 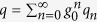) into the averaged Langevin equations (4), one can give a formal solution up to the first order for the classical part in the framework of modified Laplace transformation^36^


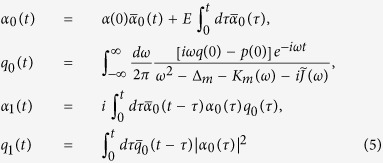


where 
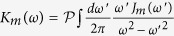
 and 
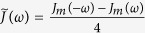
 are the real and imaginary part of the Laplace transform of the self-energy correction respectively, and the Green’s functions 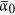 and 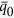 obey the Dyson equations (18) with the initial conditions 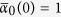, 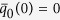 and 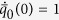
36. Base on Eq. (5), we can see that the non-vanishing intracavity field *α*_0_(∞) may induce an equilibrium position *q*_1_(∞) for the oscillator. This leads to the effective cavity detuning 
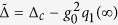
, which also alters the asymptotic dynamics of the cavity field. Accordingly, the interplay between the non-Markovian and nonlinear effects can be described more precisely in this way. Within the parameter space of our consideration, 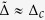, the validity of the power series assumption is guaranteed by the numerical simulations (see the Supplemental Material).

**Figure 1:**
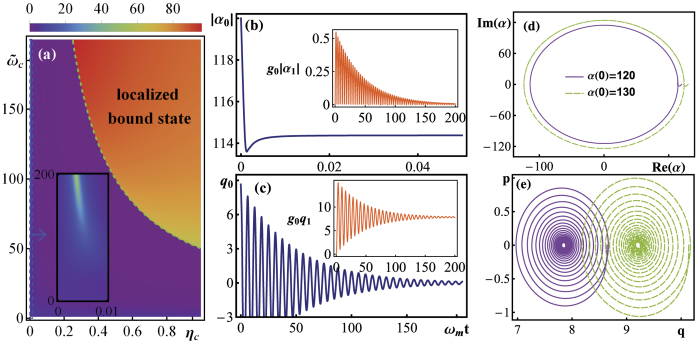
Classical dynamics in an optomechanical system, in units of *ω*_*m*_. (**a**) is the density plot of 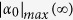, in which we show the region in the parameter space of coupling *η*_*c*_ and frequency cutoff *ω*_*c*_, where significant bound state exist with *E* = 10*ω*_*m*_ and Δ_*c*_ = 2 *ω*_*m*_. Plot (**b**–**e**) show the dynamical evolution of the classical variables, where *g*_0_ = 6 × 10^−4^*ω*_*m*_, *η*_*m*_ = 0.03, *ω*_*m*_ = 11*ω*_*m*_ and *E* = 0. The other parameters are *η*_*c*_ = 0.05, *s*_*c*_ = 3, *s*_*m*_ = 1, 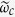  = 11*ω*_*c*_ = 1100*ω*_*m*_, *α*(0) = 120, *p*(0) = 0, and we keep 
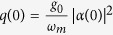
.

For general bosonic environments, the spectral density should be a Poisson-type distribution function^37^. We consider that the spectrum is of the form 
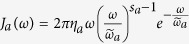
 (*a* = *c, m*), where *η*_*a*_ is a dimensionless coupling constant between the system and the environment, and 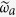 is a high-frequency cutoff^2^,^37^. The parameter *s*_*a*_ classifies the environment as sub-Ohmic (0 < *s*_*a*_ < 1), Ohmic (*s*_*a*_ = 1), and super-Ohmic (*s*_*a*_ > 1). Using the modified Laplace transformation, one can give an analytical solution for the nonequilibrium Green’s function


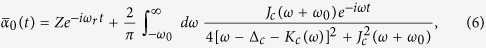


with 
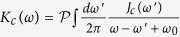
. The first term survives only when 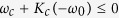, the residue 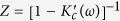 and the pole *ω*_*r*_ is located at the real *z* axis. This term corresponds to ‘localized mode’^36^,^38^, which means that the cavity field oscillates with frequency *ω*_*r*_ and does not decay. It is seem that the photons are ‘trapped’ in the cavity due to the backflow of the non-Markovian environment and do not diffuse. It is a term that determines the asymptotic dynamics of the optical field. Physically, this is equivalent to generate a bound state of the joint cavity-reservoir system^38^, which can be also determined by solving the energy eigenstates of the total Hamiltonian^39^, and such bound state is actually a stationary state with a vanishing decay rate during time evolution. The second term corresponds to nonexponential decays. In the long time limit, the bound state as well as the driving laser give rise to the non-vanishing intracavity photon numbers with 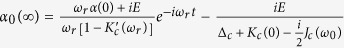. On the other hand, for the mechanical mode, however, due to the discontinuity of the self-energy correction at the real *z* axis, *q*_0_(∞) = 0. We find that the corresponding mechanical bound state can be formed only if some sharp cutoff appear in the spectral density (see section ‘Methods’ for details). Considering the feasibility, the single photon coupling *g*_0_ (key parameter) is set close to that of recently performed optomechanical experiments^35^. Figure 1(a) is the density plot of the maximum value of |*α*_0_| in the long-time limit. It maps out the regions in parameter space where localized bound state occurs. As a result, a threshold characterizing the transition from weak to strong non-Markovian regions can be defined, and it is marked by the green-dashed line, which satisfying *ω*_*c*_ + *K*_*c*_(−*ω*_0_) = 0. In the weakly non-Markovian region 0 < *η*_*c*_ < 0.01, as shown in the inset of Fig. 1(a), the red-detuned laser gives rise to a strong stationary amplitude values, which is determined by 
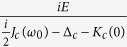
. Figure 1(b,c) show the dynamic evolution of |*α*_0_| and *q*_0_, where the first order solutions are shown in the insets. In Fig. 1(b) the optical bound state is formed when *η*_*c*_ is above the threshold, while for Fig. 1(c), the coordinate average value of the oscillator is no longer zero, which reveals that radiation pressure push the oscillator to a new equilibrium position. The evolution in the phase space is shown in Fig. 1(d,e). The limit cycles that characterize the properties of the steady state depends on the initial states, which indicates the non-Markovian property and reflects the memory effect. The initial information of the system is maintained.

We now turn to the quantum fluctuation of operators that describes the actual quantum dynamics, which are deduced as


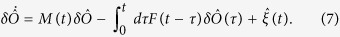


By assuming the formal solution 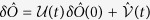, and substituting it into Eq. (7), we have


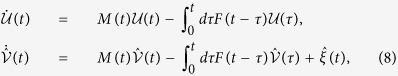


subjected to the initial conditions 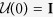 and 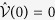. The 4 × 4 matrix


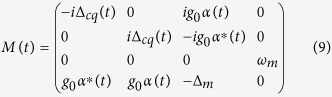


describes the linearized optomechanical coupling with non-local time-dependent classical variables, where 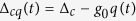 and 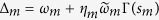. The matrix


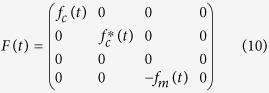


depicts the time correlation of the optomechanical system in the bosonic environments. The last term 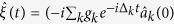, 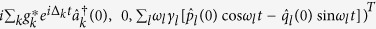 is interpreted as a noise term^23^,^24^ that depends on the initial states of the environments. It is easy to obtain the quadrature operator 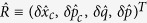 through the relation 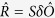, where *S* is the transformation matrix. Then the covariance matrix with components 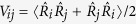 can be determined by calculating the time evolution of the second moments of the quadratures


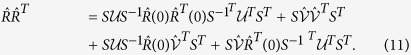


The first term is the projection of the quadratures on the system’s Hilbert space. The second term characterizes the magnitudes of the input-noise that satisfies the non-local time correlation relations^23^. The last two terms describe the effect of initial system-reservoir correlations, which had been identified as an important factor in the decoherence dynamics^40^,^41^. For the sake of simplicity, here we assume as usual the system and the reservoirs are initially uncorrelated, and the reservoirs are in thermal states. Then the noise vector 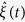 obeys the non-Markovian self-correlation 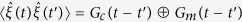, where *G*_*c*_ and *G*_*m*_ are 2 × 2 matrix


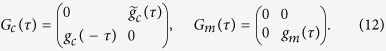


The thermal correlation functions are defined as 
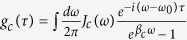
, 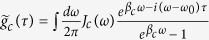 and 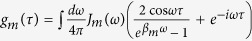, where *β* = 1/*k*_*B*_*T, k*_*B*_ is the Boltzmann constant and *T* is the initial temperature of the reservoir.

### Preservation of entanglement

**Figure 2:**
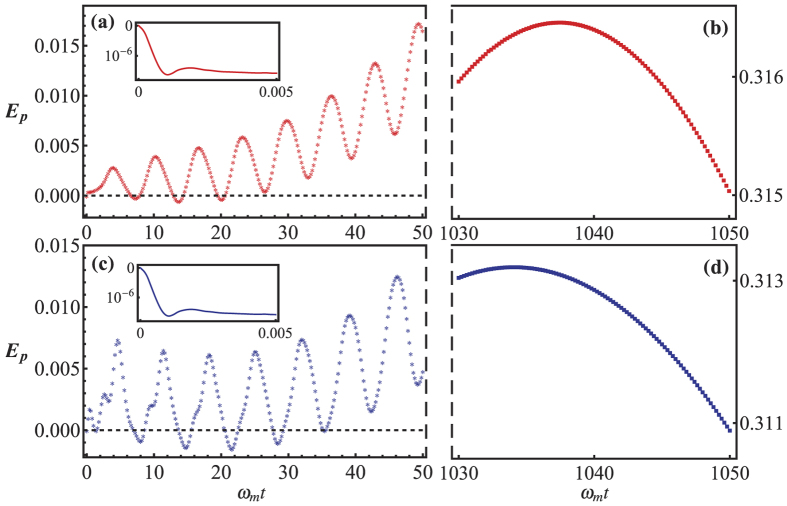
Time evolution of pseudoentanglement *E*_*p*_ in Ohmic environment. In (**a**,**b**), we keep *s*_*m*_ = 1, while in (**c**,**d**), *s*_*m*_ = 3. The other parameters are the same as Fig. 1(b) except for *η*_*m*_ = 0.8 and 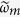  = 5*ω*_*m*_. The dynamical evolution of entanglement are shown in three regions, the initial stage of evolution *ω*_*m*_*t* < 5 × 10^−3^ shown in the insets of (**a**,**c**), the short-time scale *ω*_*m*_*t* < 50 corresponding to (**a**,**c**), the long-time scale *ω*_*m*_*t* around 1040 for (**b**,**d**). The regions *E*_*p*_ < 0 correspond to nonphysical results.

To bring quantum effects to the macroscopic level, one important way is the creation of entanglement between the optical mode and the mechanical mode. If the initial state of the system is Gaussian, then Eq. (7) will preserve the Gaussian character. The entanglement can therefore be quantified via the logarithmic negativity^42^ defined as 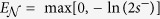, where *s*^*−*^ is the smallest symplectic eigenvalues of the partially transposed covariance matrix 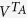. The non-Markovian effect generally lead to the nonequilibrium dynamics, therefore, we explore the optomechanical entanglement in transient regime. In order to show clearly the evolutionary path of the entanglement, we use the so-called pseudoentanglement measure^43^ defined as 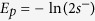, so that the logarithmic negativity of entanglement measure 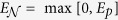. For simplicity, we assume the bath of the cavity initially in vacuum and the cavity initially in coherent state with *α*(0) = 120. The results are plotted in Fig. 2, in which 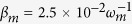 (the corresponding thermal excitation number are 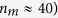. In order to show clearly the phenomenon of entanglement preservation by non-Markovianity, we shut the classical pumping field all the time by setting *E* = 0. We see in Fig. 2 that the shape of the Ohmic spectrum characterized by *s*_*m*_, has negligible impact on the initial stage of evolution (see the insets). This is because the time scale of the mechanical oscillator as well as its bath is much larger than that of the bath of the cavity, which means the non-Markovian memory effects induced by the bath of the oscillator can be ignored completely when 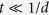, where *d* is the bandwidth of the oscillator’s bath. For the short-time and long-time scale, *s*_*m*_ slightly affect the amplitude and evolution period of the entanglement. The non-Markovian environments affect the entanglement through the bath spectrum as well as the specific type of system-reservoir interaction, which is clearly embodied in the correction functions *f*_*c*_(*t*) and *f*_*m*_(*t*), the form of which directly determines the entanglement dynamics. The Ohmic spectrum of the photon bath plays a decisive role for the time evolution of the entanglement, as it allows the existence of optical bound state. If the environmental spectral density is not a finite band, then the optical bound state is unable to form^36^, and the memory effect is not strong enough to compensate the loss of photons. Consequently the entanglement will be destroyed completely. However for the phonon bath, the Ohmic spectrum is unable to form a bound state between the mechanical mode and the environment unless some band gaps appear in the spectrum (see section ‘Methods’ for details). Therefore the resonator may only present weak non-Markovian effect, and it plays a indecisive role for the entanglement (it has limited influence on the evolution period and the value of entanglement). By changing the cutoff frequency of the phonon spectrum, we verified that the cutoff frequency do not play a major role in the entanglement dynamics, which also manifests the weak non-Markovian effects of the resonator. In addition, although the sudden death and rebirth of entanglement is also observed, it differ with the case of Markovian environment^44^ where the photons would rapidly dissipate to the memoryless environment. Here, due to the present of the bound state, the entanglement can be produced and can be preserved in non-Markovian environment after long time even without external drive. This provides a way to decoherence control of optomechanical systems, in which, a controllable quantum environment indeed have the ability to protect the quantum correlation of the internal system.

We finally discuss how to detect the generated optomechanical entanglement. Although we show the evolution of the dynamics, it still might be difficult for detection of the temporal entanglement. Fortunately, we can obtain a larger entanglement for long time, for example, *ω*_*m*_*t* around 1040 than short time *ω*_*m*_*t* < 50, which means that we can detect the entanglement at long time evolution. For measurement the logarithmic negativity, one can detect all independent entries of the covariance matrix which may be achievable by utilizing the Q-switching technology^45^. If the Q-switch is off, the composite system (i.e., the cavity and it’s environment) is closed, and the system has no output. When the Q-switch is on, the measurement can be performed by homodyning the cavity output. The duration of the Q-switched pulse should be short enough, so that it has a negligible effect on the system. The mechanical mode can be detected by employing the method put forward by Vitali *et al*.^18^, where an assistant fixed mirror and the mechanical oscillator form an additional ‘probe’ cavity mode. If this additional cavity is driven by a much weaker intracavity field so that its back-action on the mechanical mode can be neglected. In addition, the interaction between the additional cavity and it’s bath is also weak enough, so it can be well treated in the Markovian region. By adjusting parameters, the probe mode adiabatically follows the dynamics of the mechanical mode, therefore the output field gives a direct measurement of the mechanical mode. Finally the covariance matrix can be determined by changing the phases of the corresponding local oscillator and measuring the correlations between the two cavity outputs. Then one can numerically extract the logarithmic negativity.

## Conclusion

In conclusion, we have put forward a scheme to preserve the entanglement of optomechanical system in non-Markovian environment. An analytical approach for describing non-Markovian memory effects that impact on the decoherence dynamics of an optomechanical system is presented. The exact Heisenberg-Langevin equations are derived, and the perturbation solution is given in the weak single-photon coupling regime. Employing the analytical solution, we have shown that, the system dynamics change dramatically when the cavity-bath coupling strength crosses a certain threshold, which corresponds to dissipationless non-Markovian dynamics. The interplay between non-Markovian and nonlinear effects can be also explained though the perturbative method. As a quantum device which may subjected to dissipative and decoherence effects, however, our results show that the surroundings of such physical setting can protect the quantum entanglement, rather than destroy it even in the long-time scales. Our research provides a new approach to explore non-Markovian dynamics for the cavity optomechanical systems.

## Methods

### Derivation of the Heisenberg equations

Now we present a detailed derivation of the Heisenberg equations Eq. (3) given in the main text. With the total Hamiltonian 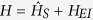, we can solve the dynamics of the optomechanical system and the reservoir in the Heisenberg representation. The system and the reservoir operators obey the equations of motion


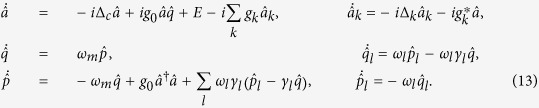


Solving Eq. (13) for 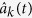, 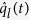 and 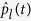



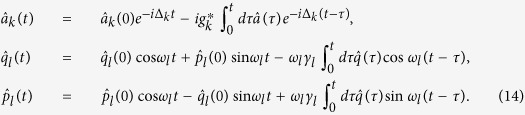


Substituting Eq. (14) into Eq. (13), we therefore obtain the integro-differential Heisenberg equations.

### The classical phase space orbits

In order to understand the classical dynamics that describes an optomechanical system embedded in memory environments, we apply a perturbative solution for Eq. (4). In realistic physical settings, the single-photon coupling strength is extremely weak, thus the perturbation expansion approach is a good approximation. The corresponding variables in the zeroth-order is given by


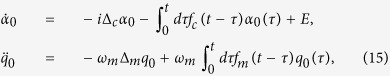


and the first-order reads


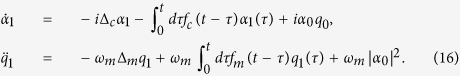


Equations (15) and (16) are exactly solvable by using the modified Laplace transformation, e.g., 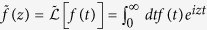. After time scaling transformation, it is easy to obtain


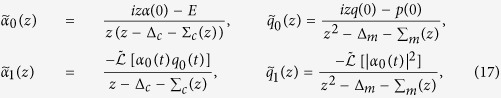


where 
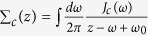
 and 
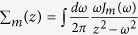
 are the Laplace transform of the self-energy correction. The modified Bromwich integral is then given by 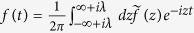. In view of Eq. (17), we define the Green’s functions 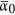 and 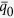, which obey the Dyson equations


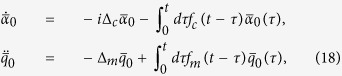


and is subjected to the initial conditions 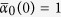, 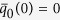 and 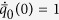. The solution of 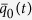 is given by 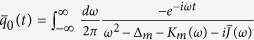, while 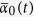 is given in Eq. (6).

The dissipationless non-Markovian dynamics usually requires 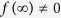 (in absence of any external driving). In other word, it is referred to a process of nonthermal stabilization^46^, in which the initial information of the system partially maintains. In this case, there should be poles exist on the real *z* axis for 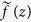. Due to the discontinuity of the Laplace transform of the self-energy correction, however, the real poles exist only in the frequency regions that all spectral density vanishes. In this region, 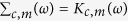. From the physical point of view, it can be explained by the bound state generated between the system and its environment^47^,^48^,^49^, which is actually a stationary state with a vanishing decay rate. It usually occurs when the environments has band gaps or a finite band.

The reservoir and its coupling to the system are fully characterized by the spectral density *J*(*ω*). For thermal bosonic (photon and phonon) baths, the most general spectral density as introduced in^37^ should be a Poisson-type distribution function with some frequency cutoff. From the experimental point of view, it is often not possible to model the environment in an accurate way, because their density of states is unknown. However one can in principle extract the information of the spectral density of the heat baths by ingenious experiment. Recently, this work is accomplished by Gröblacher *et al*.^29^. The main experimental results show that the specific geometry of the slab cause strongly sub-Ohmic spectral densities. To theoretically explore the decoherence dynamics, we therefore consider the Ohmic spectrum.

On the other hand, as discussed above, the imaginary part of 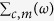 is discontinuous on the real *z* axis, i.e., 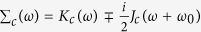 and 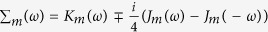. For the cavity mode, the corresponding pole is determined by 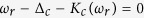 with 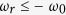. For the mechanical mode, the imaginary part of 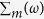 vanishes only at the zero point, although an Ohmic spectrum with a finite band is considered. The main reason lies in their different internal memory kernels, the structure of which is entirely determined by the specific type of interactions between the system components and the corresponding environment. For the zero point, note that 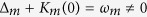. Thus it is unable to form a bound state between the mechanical mode and the environment unless some band gaps appear in the spectrum^38^.

## Additional Information

**How to cite this article**: Cheng, J. *et al*. Preservation Macroscopic Entanglement of Optomechanical Systems in non-Markovian Environment. *Sci. Rep.* **6**, 23678; doi: 10.1038/srep23678 (2016).

## Supplementary Material

Supplementary Information

## References

[b1] Breuer H. P. & Petruccione F. The Theory of Open Quantum Systems. Ch. 3, 109–216 (Oxford University Press, Oxford, 2002).

[b2] Weiss U. Quantum Dissipative Systems 3rd ed. Ch. 7, 93–99 (World Scientific Press, Singapore, 2008).

[b3] DiVincenzo D. P. Real and realistic quantum computers. Nature 393, 113–114 (1998).

[b4] Knill E., Laflamme R. & Milburn G. J. A scheme for efficient quantum computation with linear optics. Nature 409, 46–52 (2001). 11343107 10.1038/35051009

[b5] Verstraete F., Wolf M. M. & Cirac J. I. Quantum computation and quantum-state engineering driven by dissipation. Nat. Phys. 5, 633–636 (2009).

[b6] Kastoryano M. J., Wolf M. M. & Eisert J. Precisely timing dissipative quantum information processing. Phys. Rev. Lett. 110, 110501 (2013). 25166517 10.1103/PhysRevLett.110.110501

[b7] Chruściński D. & Kossakowski A. Non-Markovian quantum dynamics: Local versus nonlocal. Phys. Rev. Lett. 104, 070406 (2010). 20366866 10.1103/PhysRevLett.104.070406

[b8] Xu J. S. . Experimental demonstration of photonic entanglement collapse and revival. Phys. Rev. Lett. 104, 100502 (2010). 20366406 10.1103/PhysRevLett.104.100502

[b9] Liu B. H. . Experimental control of the transition from Markovian to non-Markovian dynamics of open quantum systems. Nat. Phys. 7, 931–934 (2011).

[b10] Chin A. W., Huelga S. F. & Plenio M. B. Quantum metrology in non-Markovian environments. Phys. Rev. Lett. 109, 233601 (2012). 23368199 10.1103/PhysRevLett.109.233601

[b11] Deffner S. & Lutz E. Quantum speed limit for non-Markovian dynamics Phys. Rev. Lett. 111, 010402 (2013). 23862985 10.1103/PhysRevLett.111.010402

[b12] Reich D. M., Katz N. & Koch C. P. Exploiting non-Markovianity for quantum control. Sci. Rep. 5, 12430. 26199059 10.1038/srep12430 PMC4510487

[b13] Rivas Á., Huelga S. F. & Plenio M. B. Entanglement and non-Markovianity of quantum evolutions. Phys. Rev. Lett. 105, 050403 (2010). 20867898 10.1103/PhysRevLett.105.050403

[b14] Breuer H. P., Laine E. M. & Piilo J. Measure for the degree of non-Markovian behavior of quantum processes in open systems. Phys. Rev. Lett. 103, 210401 (2009). 20366019 10.1103/PhysRevLett.103.210401

[b15] Vasile R., Maniscalco S., Paris M. G. A., Breuer H. P. & Piilo J. Quantifying non-Markovianity of continuous-variable Gaussian dynamical maps. Phys. Rev. A 84, 052118 (2011).

[b16] Lorenzo S., Plastina F. & Paternostro M. Geometrical characterization of non-Markovianity. Phys. Rev. A 88, 020102(R) (2013).

[b17] Chruściński D. & Maniscalco S. Degree of non-Markovianity of quantum evolution. Phys. Rev. Lett. 112, 120404 (2014). 24724632 10.1103/PhysRevLett.112.120404

[b18] Vitali D. . Optomechanical entanglement between a movable mirror and a cavity field. Phys. Rev. Lett. 98, 030405 (2007). 17358666 10.1103/PhysRevLett.98.030405

[b19] Kippenberg T. J. & Vahala K. J. Cavity optomechanics: Back-action at the mesoscale. Science 321, 1172–1176 (2008). 18755966 10.1126/science.1156032

[b20] Gröblacher S., Hammerer K., Vanner M. R. & Aspelmeyer M. Observation of strong coupling between a micromechanical resonator and an optical cavity field. Nature 460, 724–727 (2009). 19661913 10.1038/nature08171

[b21] O’Connell A. D. . Quantum ground state and single-phonon control of a mechanical resonator. Nature 464, 697–703 (2010). 20237473 10.1038/nature08967

[b22] Thompson J. D. . Strong dispersive coupling of a high-finesse cavity to a micromechanical membrane. Nature 452, 72–75 (2008). 18322530 10.1038/nature06715

[b23] Giovannetti V. & Vitali D. Phase-noise measurement in a cavity with a movable mirror undergoing quantum Brownian motion. Phys. Rev. A 63, 023812 (2001).

[b24] Genes C., Vitali D., Tombesi P., Gigan S. & Aspelmeyer M. Ground-state cooling of a micromechanical oscillator: Comparing cold damping and cavity-assisted cooling schemes. Phys. Rev. A 77, 033804 (2008).

[b25] Agarwal G. S. & Huang S. Electromagnetically induced transparency in mechanical effects of light. Phys. Rev. A 81, 041803(R) (2010).

[b26] Rabl P. Photon blockade effect in optomechanical systems. Phys. Rev. Lett. 107, 063601 (2011). 21902322 10.1103/PhysRevLett.107.063601

[b27] Bayindir M., Temelkuran B. & Ozbay E. Tight-binding description of the coupled defect modes in three-dimensional photonic crystals. Phys. Rev. Lett. 84, 2140 (2000). 11017228 10.1103/PhysRevLett.84.2140

[b28] Hartmann M. J., Brandão F. G. S. L. & Plenio M. B. Strongly interacting polaritons in coupled arrays of cavities. Nat. Phys. 2, 849–855 (2006).

[b29] Gröblacher S. . Observation of non-Markovian micromechanical Brownian motion. Nat. Commun. 6, 7606 (2015). 26216619 10.1038/ncomms8606 PMC4525213

[b30] Wu M. H., Lei C. U., Zhang W. M. & Xiong H. N. Non-Markovian dynamics of a microcavity coupled to a waveguide in photonic crystals. Opt. Express 18, 18407–18418 (2010). 20721235 10.1364/OE.18.018407

[b31] Ford G. W., Lewis J. T. & O’Connell R. F. Quantum Langevin equation. Phys. Rev. A 37, 4419–4428 (1988). 10.1103/physreva.37.4419 9899572

[b32] Chan J. . Laser cooling of a nanomechanical oscillator into its quantum ground state. Nature 478, 89–92 (2011). 21979049 10.1038/nature10461

[b33] Teufel J. D. . Sideband cooling of micromechanical motion to the quantum ground state. Nature 475, 359–363 (2011). 21734657 10.1038/nature10261

[b34] Arcizet O., Cohadon P. F., Briant T., Pinard M. & Heidmann A. Radiation-pressure cooling and optomechanical instability of a micromirror. Nature 444, 71–74 (2006). 17080085 10.1038/nature05244

[b35] Aspelmeyer M., Kippenberg T. J. & Marquardt F. Cavity optomechanics. Rev. Mod. Phys. 86, 1391–1452 (2014).

[b36] Zhang W. M., Lo P. Y., Xiong H. N., Tu M. W. Y. & Nori F. General non-Markovian dynamics of open quantum systems. Phys. Rev. Lett. 109, 170402 (2012). 23215166 10.1103/PhysRevLett.109.170402

[b37] Leggett A. J. . Dynamics of the dissipative two-state system. Rev. Mod. Phys. 59, 1–85 (1987).

[b38] Cheng J., Zhang W. Z., Han Y. & Zhou L. Robust fermionic-mode entanglement of a nanoelectronic system in non-Markovian environments. Phys. Rev. A 91, 022328 (2015).

[b39] Tong Q. J., An J. H., Luo H. G. & Oh C. H. Mechanism of entanglement preservation. Phys. Rev. A 81, 052330 (2010).

[b40] Romero L. D. & Paz J. P. Decoherence and initial correlations in quantum Brownian motion. Phys. Rev. A 55, 4070–4083 (1997).

[b41] Dijkstra A. G. & Tanimura Y. Non-Markovian entanglement dynamics in the presence of system-bath coherence. Phys. Rev. Lett. 104, 250401 (2010). 20867350 10.1103/PhysRevLett.104.250401

[b42] Adesso G. & Illuminati F. Gaussian measures of entanglement versus negativities: Ordering of two-mode Gaussian states. Phys. Rev. A 72, 032334 (2005).

[b43] Wang G., Huang L., Lai Y. C. & Grebogi C. Nonlinear dynamics and quantum entanglement in optomechanical systems. Phys. Rev. Lett. 112, 110406 (2014). 24702337 10.1103/PhysRevLett.112.110406

[b44] Yu T. & Eberly J. H. Sudden death of entanglement. Science 323, 598–601 (2009). 19179521 10.1126/science.1167343

[b45] Früngel F. B. A. Optical Pulses-Lasers-Measuring Techniques. Ch. 1, 192 (Academic Press, London, 2014).

[b46] Xiong H. N., Lo P. Y., Zhang W. M., Feng D. H. & Nori F. Non-Markovian complexity in the quantum-to-classical transition. Sci. Rep. 5, 13353 (2015). 26303002 10.1038/srep13353 PMC4548183

[b47] John S. & Quang T. Spontaneous emission near the edge of a photonic band gap. Phys. Rev. A 50, 1764–1769 (1994). 9911069 10.1103/physreva.50.1764

[b48] Lodahl P. . Controlling the dynamics of spontaneous emission from quantum dots by photonic crystals. Nature 430, 654–657 (2004). 15295594 10.1038/nature02772

[b49] Bellomo B., Franco R. L., Maniscalco S. & Compagno G. Entanglement trapping in structured environments. Phys. Rev. A 78, 060302(R) (2008).

