# Supplementary material for: Preservation Macroscopic Entanglement of Optomechanical Systems in non-Markovian Environment
Source: Sci Rep. 2016 Apr 1;6:23678. doi: 10.1038/srep23678 (PMC4817058; doi:10.1038/srep23678)
Supplement: Supplementary Information [file srep23678-s1.pdf]

Supplementary Material for

"Preservation Macroscopic Entanglement of Optomechanical Systems in non-Markovian Environment"

Jiong Cheng,[1] Wen-Zhao Zhang,[1] Ling Zhou*,[1] and Weiping Zhang[2]

[1]School of Physics and Optoelectronic Technology,
Dalian University of Technology, Dalian 116024, People's Republic of China
[2]State Key Laboratory of Precision Spectroscopy, Department of Physics,
East China Normal University, Shanghai 200062, People's Republic of China

I. THE CLASSICAL PHASE SPACE ORBITS

To test the validity of the perturbation expansion method, we numerically simulate Eqs. (4). The results are presented in Fig. S1, which agree very well with the analytical solution given by Eqs. (5). Here we take $g_0/\omega_m = 6 \times 10^{-4}$ (all the parameters are the same as chosen in Figs. 1(b) and 1(c)). For most of the cavity-optomechanics experiments, the single-photon optomechanical coupling rate is even weaker [1], i.e., $g_0/\omega_m \lesssim 10^{-4}$. In this region, one can safely apply the the power series assumption, which gives an accurate description of the classical dynamics.

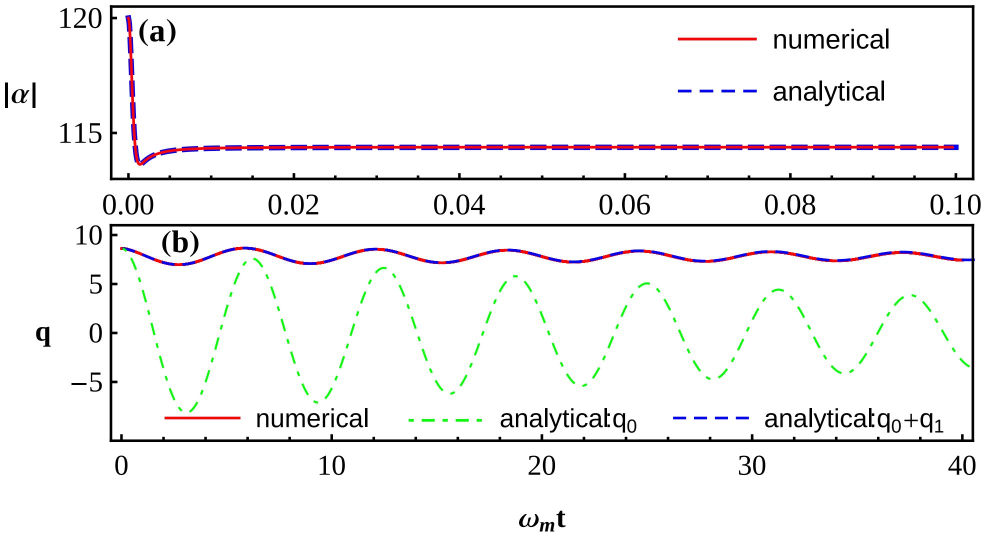

FIG. S1: (Color online) Dynamical evolution of the classical variables. The parameters are the same as that given in Fig. 1(b) and 1(c)

II TRANSITION FROM NON-MARKOVIAN TO MARKOVIAN DYNAMICS

Now we study how the strength of non-Markovian memory effects impact on the decoherence dynamics of the optomechanical system. To this end, we discuss the transition from non-Markovian to Markovian dynamics. The strength of non-Markovian memory effect is highly dependent on the structure of the reservoir as well as the interaction strength between the system and reservoir. By decreasing the dimensionless coupling constant η_c, the non-Markovianity of the system decreases. This corresponds to three different regions: strong non-Markovianity, weak non-Markovianity and the truly Markovian region. Fig. S2 shows the dynamical evolution of the classical variables in different regions. In the strong non-Markovian region (orange solid line), the bound state is formed, which exhibits periodic oscillations as ω_r is a real number (see Eq.(6) or Fig.1d). The initial state information of the system partially maintains in this case. If the coupling constant is less than the critical value, the dissipation process in terms of a profile of exponential-like decay accompanied with short-time oscillations, which is a manifestation of weak non-Markovian memory effects (green dashed and blue dotted line). The initial state information, however, is

lost (with or without the driving field). In the Born-Markovian approximation, the time correlation function of the environment should be a delta function and the system-reservoir coupling is very weak, therefore, the environment is memoryless. The dissipation process present exponential decay (purple dot-dashed line).

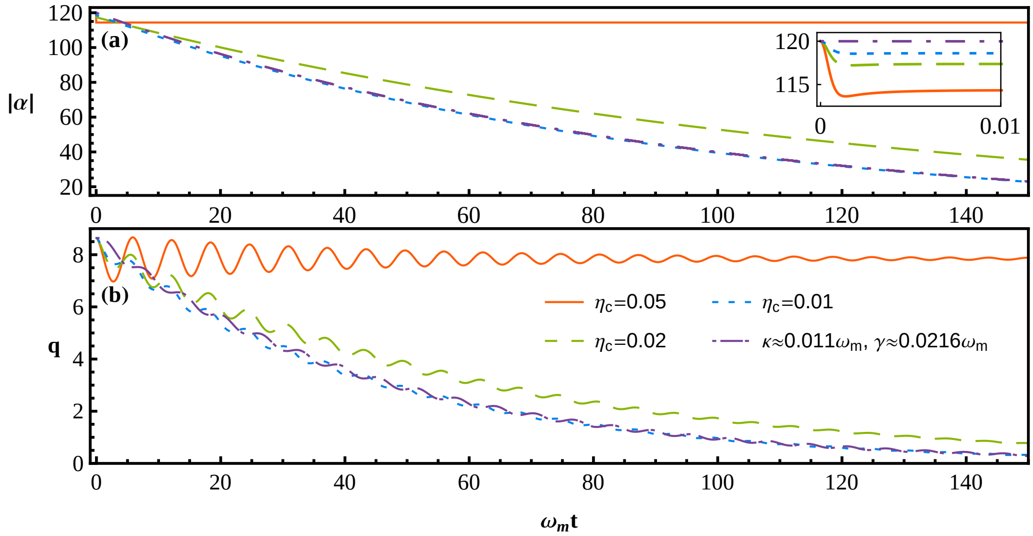

FIG. S2: (Color online) Dynamical evolution of the classical variables from non-Markovian to Markovian regions. By decreasing the dimensionless coupling constant η_c, the non-Markovianity of the system decreases. The chosen parameters correspond to strong non-Markovianity: $\eta_c = 0.05$, weak non-Markovianity: $\eta_c = 0.02$, $\eta_c = 0.01$ and the truly Markovian region: $\kappa \approx 0.011\omega_m$, $\gamma \approx 0.0216\omega_m$, where κ and γ are the decay rate of the cavity and mechanical modes respectively. The other parameters are the same as Fig. 1(b).

[1] Aspelmeyer M., Kippenberg T. J., & Marquardt F. Cavity optomechanics. *Rev. Mod. Phys.* **86**, 1391-1452 (2014).
